# Formononetin Administration Ameliorates Dextran Sulfate Sodium-Induced Acute Colitis by Inhibiting NLRP3 Inflammasome Signaling Pathway

**DOI:** 10.1155/2018/3048532

**Published:** 2018-01-08

**Authors:** Dacheng Wu, Keyan Wu, Qingtian Zhu, Weiming Xiao, Qing Shan, Zhigang Yan, Jian Wu, Bin Deng, Yan Xue, Weijuan Gong, Guotao Lu, Yanbing Ding

**Affiliations:** ^1^Department of Gastroenterology, Affiliated Hospital of Yangzhou University, Yangzhou University, Yangzhou, China; ^2^Laboratory of Gastroenterology, Affiliated Hospital of Yangzhou University, Yangzhou University, Yangzhou, China; ^3^Department of Immunology, School of Medicine, Yangzhou University, Yangzhou, China

## Abstract

Formononetin is a kind of isoflavone compound and has been reported to possess anti-inflammatory properties. In this present study, we aimed to explore the protective effects of formononetin on dextran sulfate sodium- (DSS-) induced acute colitis. By intraperitoneal injection of formononetin in mice, the disease severity of colitis was attenuated in a dose-dependent manner, mainly manifesting as relieved clinical symptoms of colitis, mitigated colonic epithelial cell injury, and upregulations of colonic tight junction proteins levels (ZO-1, claudin-1, and occludin). Meanwhile, our study found that formononetin significantly prevented acute injury of colonic cells induced by TNF-*α* in vitro, specifically manifesting as the increased expressions of colonic tight junction proteins (ZO-1, claudin-1, and occludin). In addition, the result showed that formononetin could reduce the NLRP3 pathway protein levels (NLRP3, ASC, IL-1*β*) in vivo and vitro, and MCC950, the NLRP3 specific inhibitor, could alleviate the DSS-induced mice acute colitis. Furthermore, in the foundation of administrating MCC950 to inhibit activation of NLRP3 inflammasome, we failed to observe the protective effects of formononetin on acute colitis in mice. Collectively, our study for the first time confirmed the protective effects of formononetin on DSS-induced acute colitis via inhibiting the NLRP3 inflammasome pathway activation.

## 1. Introduction

Inflammatory bowel disease (IBD) is a kind of noninfectious inflammatory disease, mainly consisting of ulcerative colitis (UC) and Crohn's disease (CD). Epidemiological data showed that IBD has become a global problem, the incidence of IBD in high-risk areas remained stable in these years, while in low-risk areas such as southern Europe, Asia, and most developing countries, the incidence is increasing [1].

The specific mechanism of IBD is still unclear, while there is no doubt that intestinal mucosal immune dysfunction plays an important role in the pathogenesis of IBD [2, 3]. Immunosuppressors, such as 6-mercaptopurine, azathioprine, methotrexate, and cyclosporin A, which could block the abnormal immune activation, are often recommended to treat IBD patients in the clinical setting [4, 5]. Nevertheless, this kind of immunosuppressors could affect the balance of normal immune system and even cause severe adverse effects, including myelosuppression, infection, and liver damage. Hence, seeking for novel immunomodulatory drugs that could effectively alleviate mucosal inflammation with minimum or no side effects seems to be vital in the clinical prevention and treatment of IBD.

Formononetin is a kind of isoflavone compound and widely exists in the natural plants. It is one of the major biologically active compounds in a variety of Chinese medicinal herbs, such as astragalus, which has been traditionally used for treatment of diabetes over 2000 years in China. Current research evidences indicated that formononetin played several different effects, such as, anti-inflammatory [6, 7], antioxidative [8], antitumor [9], and promoting apoptosis [10]. Lima Cavendish et al. confirmed that formononetin has an anti-inflammatory effect on rats with peritonitis [6] and could protect mice from lipopolysaccharide-induced acute lung injury [7]. However, the specific effects of formononetin on acute colitis have not been well defined and documented.

Inflammasome, a key component in host inflammation regulation, was put forward by Tschopp research team for the first time in 2002 [11]. The inflammasome is a cytosolic multimeric protein complex composed of nucleotide-binding domain and leucine-rich repeat-containing proteins (NLRs) or AIM2, adaptor protein apoptosis-associated speck-like protein containing CARD (ASC), and cysteine-aspartic acid protease- (caspase-) 1. There has been found four kinds of inflammasome: NLRP1, NLRP3, NLRC4, and AIM2 [12–14], among which NLRP3 inflammasome is the most thoroughly studied. Previous study had shown that NLRP3 inflammasome may play a critical role in the pathogenesis of IBD. Clinical research indicated that the susceptibility to CD may be related to NLRP3 gene polymorphism [15], in a study of Chinese population (232 CD cases, 56 UC cases, and 247 healthy control cases), the results indicated that NLRP3 gene expression was not significantly different between IBD group and control group; There were no significant variations in NLRP3 gene expression in the CD subgroup analysis; While in the UC subgroup analysis, the two mutations of SNPS-rs10754558 and rsl0925019 on NLRP3 gene were associated with the incidence of UC, demonstrating that NLRP3 gene played an important role in the pathogenesis of UC in Chinese patients [16]. In an acute colitis model in mice induced by dextran sulfate sodium (DSS), NLRP3 gene knockout or medical inhibition of the NLRP3 inflammasome activation both exerted protective effects on mice [17, 18]. However, whether formononetin had a regulatory effect on NLRP3 inflammasome pathway remained still unclear. In this study, we aimed to explore the protective effects of formononetin on DSS-induced acute colitis and investigate the potential underlying mechanism.

## 2. Materials and Methods

### 2.1. Mice and Reagents

Eight- to ten-week-old male C57BL/6 mice were purchased from Comparative Medicine Centre of Yangzhou University (Yangzhou, Jiangsu Province, China). All mice were housed in a specific pathogen-free (SPF) standard room under 12/12 h light-dark cycle at 26°C, relative humidity 45%, given water ad libitum, fed standard laboratory chow, and were allowed to acclimatize to the new environment for at least 1 week. All methods were carried out in accordance with the principles of laboratory animal care (NIH publication Number 85Y23, revised 1996), and all experimental protocols were approved by the experimental animal ethics committee of Affiliated Hospital of Yangzhou University.

Formononetin (F141481) was purchased from Aladdin Bio-Chem Technology company (Aladdin, Shanghai, China), and DSS was purchased from MP Biomedical company (MP Biomedical, Santa Ana, CA, USA). The primary antibodies: myeloperoxidase (MPO, ab9535), cluster of differentiation 68 (CD68, ab955), claudin-1 (ab15098), occludin (ab31721), zonula occludens-1 (ZO-1, ab59720), interleukin-1*β* (IL-1*β*, ab9722), and GAPDH (ab8245) were purchased from Abcam company (Abcam, Cambridge, UK), anti-NLRP3 (15101) antibody was purchased from CST company (Cell Signaling Technology, Boston, USA), and anti-ASC (sc-22514-R) antibody was purchased from Santa Cruz Biotechnology (Santa Cruz, CA, USA). Goat anti-rabbit and rabbit anti-mouse secondary antibodies were purchased from Abcam company (Abcam, Cambridge, UK). Recombinant human tumor necrosis factor-alpha (TNF-*α*) was purchased from Cayman company (Cayman, Michigan, USA). MTS (3-(4,5-dimethylthiazol-2-yl)-5-(3-carboxymethoxyphenyl)-2-(4-sulfophenyl)-2H-tetrazolium) assay was purchased from Promega company (Promega, Madison, WI, USA).

### 2.2. Experimental Design and Procedures In Vivo

Experimental mice were randomly assigned to five groups (*n* = 8 per group): vehicle, DSS, low-dose formononetin (25 mg/kg, L-dose), middle-dose formononetin (50 mg/kg, M-dose), and high-dose formononetin (100 mg/kg, H-dose) group. Except the vehicle group, mice in the other four groups were given DSS solution (2.5%, dissolved in distilled water) ad libitum to induce the acute colitis model; meanwhile, the vehicle group was administered distilled water equivalently. Mice in the drug intervention groups (L-dose, M-dose, and H-dose group) were daily injected with formononetin (dissolved in 5% DMSO) intraperitoneally, while the vehicle group and DSS model group were administered 5% DMSO in the same way.

To inhibit the NLRP3 inflammasome in vivo, mice were treated with MCC950 (50 mg/kg, *n* = 8) intraperitoneally (the mice in the control group were treated with PBS, *n* = 8) every other day from one day before to day 7 of DSS administration [18]. Meanwhile, high-dose formononetin (100 mg/kg) was treated daily on day 1–8 of DSS administration (*n* = 8) to identify the underlying molecular mechanisms of formononetin on acute colitis.

The severity of acute colitis was judged by measuring body weight, stool consistency, and the occurrence of gross blood in the stool in mice every day, and the disease activity index (DAI) score was also used to assess the disease severity of colitis. DAI score was calculated by grading on a scale of 0 to 4 using the following parameters [19]: loss of body weight (0, normal; 1, 0–5%; 2, 5–10%; 3, 10–15%; and 4, >15%), stool consistency (0, normal; 2, loose stool; and 4, watery diarrhea), and the occurrence of gross blood in the stool (0, negative; 2, slight bleeding; and 4, gross bleeding). The combined DAI was scored by two independent investigators.

Nine days after DSS administration, animals were anaesthetized with the intraperitoneal administration of sodium pentobarbital (50 mg/kg) then sacrificed; their distal colonic tissues were dissected immediately. A portion of the colonic tissues was fixed for histological analysis and the rest were stored at −80°C for further investigation. Blood samples were obtained from the tail veins and stored at −80°C for analysis.

### 2.3. Histological Examination

Distal colonic tissues were fixed in 4% paraformaldehyde (dissolved in PBS) and embedded in paraffin and stained with hematoxylin and eosin. Two investigators who were blind to the experimental treatment scored the degree of colonic injury by light microscopy. The severity of colonic injury was evaluated by the following parameters [20]: epithelial damage (0, normal; 1, minimal loss of goblet cells; 2, extensive loss of goblet cells; 3, minimal loss of crypts and extensive loss of goblet cells; and 4, extensive loss of crypts); and infiltration (0, normal; 1, infiltrate around crypt bases; 2, infiltrate in muscularis mucosa; 3, extensive infiltrate in muscularis mucosa with edema; and 4, infiltration of submucosa).

### 2.4. Cell Culture and Treatment In Vitro

Human colon carcinoma cell line HCT-116 was purchased from Shanghai cell bank of Chinese Academy of Sciences (Shanghai, China) and used for in vitro experiment. HCT-116 cells were cultured in Dulbecco's modified Eagle medium (DMEM) supplemented with 10% fetal bovine serum at 37°C with a humidified atmosphere containing 5% CO_2_. TNF-*α* (100 ng/ml) was used to establish a cell injury model [21], and the HCT-116 cells were incubated with different concentrations of formononetin (25 *μ*M, 50 *μ*M) or 0.1% DMSO (vehicle) to verify the protective effect of formononetin. Twelve hours after formononetin intervention, cellular protein was extracted and stored for further experiments.

Cell viability was evaluated by using the MTS assay. After 1 h of adhesion of HCT-116 cells, different doses of formononetin (dissolved in DMSO) or TNF-*α* (dissolved in PBS) were plated in 96-well plates and wells containing only HCT-116 (0.1% DMSO or PBS in complete medium) were used as control groups. Twelve hours later, cells were treated with MTS in accordance with the manufacturer's instructions.

### 2.5. Immunofluorescence (IF) and Western Blot (WB)

IF and WB analyses were carried out as previously described [22, 23]. Briefly, for IF analysis, slides were incubated overnight at 4°C in a humid chamber with an antibody against MPO (1 : 500 dilution), CD68 (1 : 200 dilution), claudin-1 (1 : 200 dilution), occludin (1 : 200 dilution), ZO-1 (1 : 200 dilution), and NLRP3 (1 : 200 dilution) and then incubated by biotinylated secondary antibody (1 : 500 dilution) for 60 minutes. For WB analysis, the polyvinylidene difluoride (PVDF) membranes were blocked by 5% (*w*/*v*) bovine serum albumin in Tris-buffered saline/0.05% tween-20 (TBST) at room temperature for 2 h in a covered container and incubated overnight at 4°C with primary antibodies against NLRP3 (1 : 1000 dilution), ASC (1 : 1000 dilution), IL-1*β* (1 : 1000 dilution), claudin-1 (1 : 1000 dilution), occludin (1 : 1000 dilution), ZO-1 (1 : 1000 dilution), and GAPDH (1 : 2000 dilution) in blocking buffer. On the next day, membranes were washed with TBST (3∗10 min) and incubated with a secondary goat anti-mouse or goat anti-rabbit IgG horseradish peroxidase (HRP) antibody (1 : 10,000 dilution) diluted in 5% (*w*/*v*) dry nonfat milk in TBST for 1 h at room temperature. Finally, membranes were washed with TBST (3∗10 minutes), developed by using the ECL detection system (Santa Cruz Biotechnology), quickly dried, and exposed to ECL film.

### 2.6. Enzyme-Linked Immunosorbent Assay (ELISA)

ELISA analyses were carried out as previously described [24]. Briefly, the colon tissues were homogenated in PBS and then carried out by centrifugation (12000 rpm, 4°C, 30 min) to obtain supernatant. The TNF-*α* and IL-1*β* levels were measured with the commercial kits (Affymetrix ebioscience, Santiago, USA).

### 2.7. Statistical Analysis

Statistical analysis was performed by SPSS 22.0 software. Results are presented as mean ± standard deviation (SD). The Kruskal–Wallis test followed by the Mann–Whitney *U* test was used to evaluate the differences in histopathological scores. Statistical analysis was performed using one-way ANOVA followed by the Student–Newman–Keuls test as a post hoc test. A value of *p* < 0.05 was considered statistically significant.

## 3. Results

### 3.1. Formononetin Protected against DSS-Induced Acute Colitis in Mice

**Figure 1:**
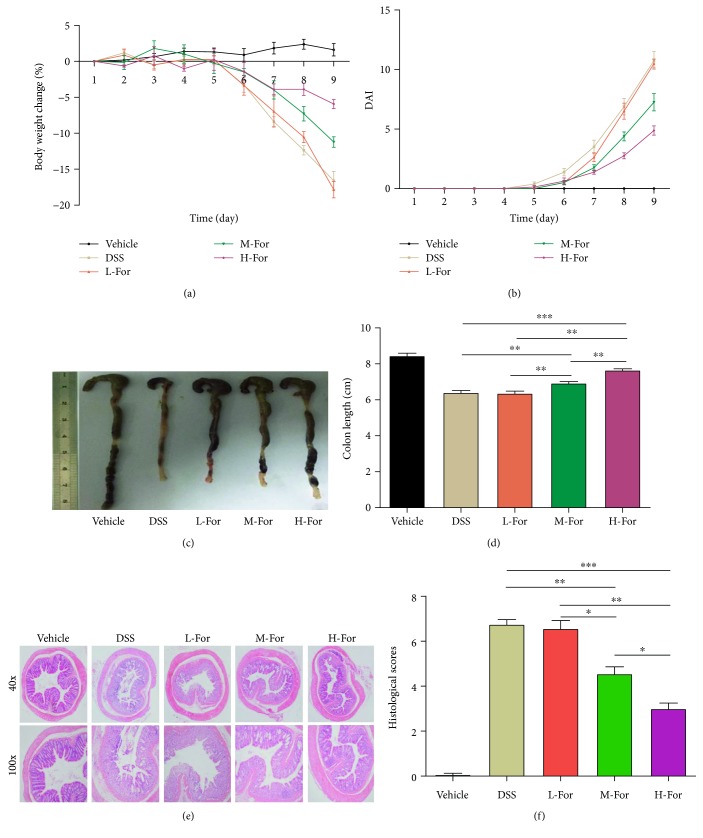
Formononetin (For) attenuates dextran sulfate sodium- (DSS-) induced acute colitis in mice. (a) Body weights of mice and (b) disease activity index (DAI) during the disease process, (c) morphological changes in the mice colons, (d) variations of colon length of mice, (e) representative HE staining, and (f) histological scores of colonic tissue. ^∗^ *p* < 0.05, ^∗∗^ *p* < 0.01, and ^∗∗∗^ *p* < 0.001.

As expected, colonic injury, weight loss, bloody stool, and watery diarrhea were observed in mice after DSS feeding. After formononetin administration, we observed that formononetin could alleviate the clinical symptoms of acute colitis mice in a dose-dependent manner. The body weight of mice in DSS group decreased by 15%, in contrast, the body weight of mice in H-dose administration group decreased by 6.7% after nine days DSS feeding (Figure 1(a)); meanwhile, the DAI scores were 11 and 4.5 in two groups, respectively. (Figure 1(b)). Then, we further examined the degrees of colonic histopathological injury to assess the disease severity of colitis and found that the histopathological manifestations in vehicle group presented as a normal colonic appearance, while in DSS group, the histopathological characteristics were displayed as colonic epithelial cell injury and a great number of inflammatory cells infiltrating into the mucosa and the submucosa of colon; in contrast, H-dose formononetin (100 mg/kg) markedly alleviated the histological features of colonic mucosa damage, characterized as lower degree of epithelial cell injury and inflammatory infiltration. Additionally, the protective effects of formononetin on acute colitis appeared to be dose-dependent and we failed to confirm the protective effects of formononetin in L-dose group (25 mg/kg) (Figures 1(e) and 1(f)). Furthermore, as for the changes in the average length for colon after formononetin treatment (Figures 1(c) and 1(d)), the changing trend was consistent with the results of histopathological manifestations.

### 3.2. Formononetin Reduced Colonic Inflammation in Mice

**Figure 2:**
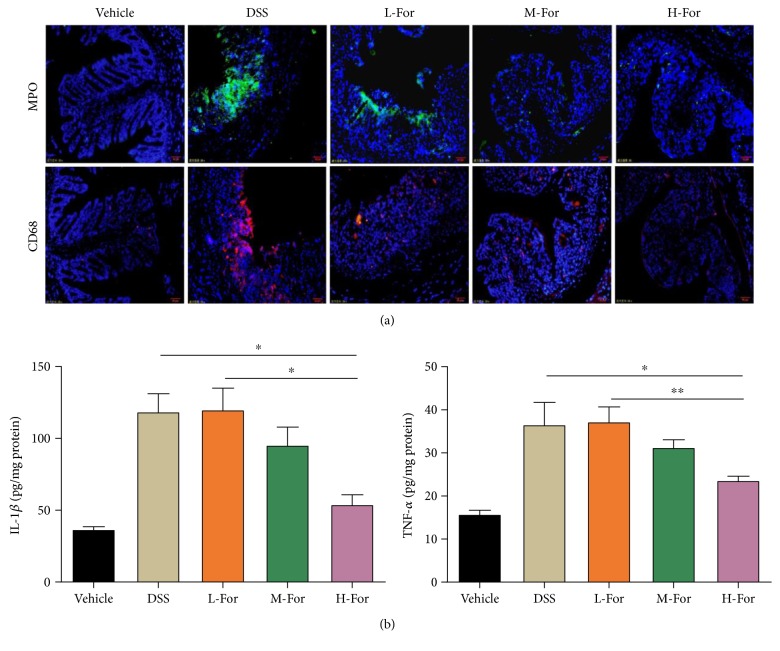
Effect of For on the colonic inflammation in mice. (a) Representative immunofluorescence images for MPO (Green) and CD68 (Red) in the colonic tissue. (b) The level of inflammatory cytokines in the colonic tissue. ^∗^ *p* < 0.05 and ^∗∗^ *p* < 0.01.

Neutrophil and macrophage infiltrations could be viewed as evaluative parameters for disease severity in acute colitis mice. As Figure 2(a) showed, the MPO and CD68 staining results were positive in mice with colitis, indicating a great number of neutrophils and macrophage infiltrating into the colonic tissues. After formononetin administration, there were fewer infiltrating neutrophils and macrophages in injured colonic tissue. In addition, the level of inflammatory cytokines in the colon of mice was detected by ELISA method to assess the severity of acute colitis; as expected, the levels of TNF-*α* and IL-1*β* were significantly decreased after formononetin treatment (Figure 2(b)). The results of inflammatory infiltration and inflammatory cytokine expression turned out to be consistent with the pathological results.

### 3.3. Formononetin Relieved DSS-Induced Colonic Epithelial Tight Junction Disruption in Mice

**Figure 3:**
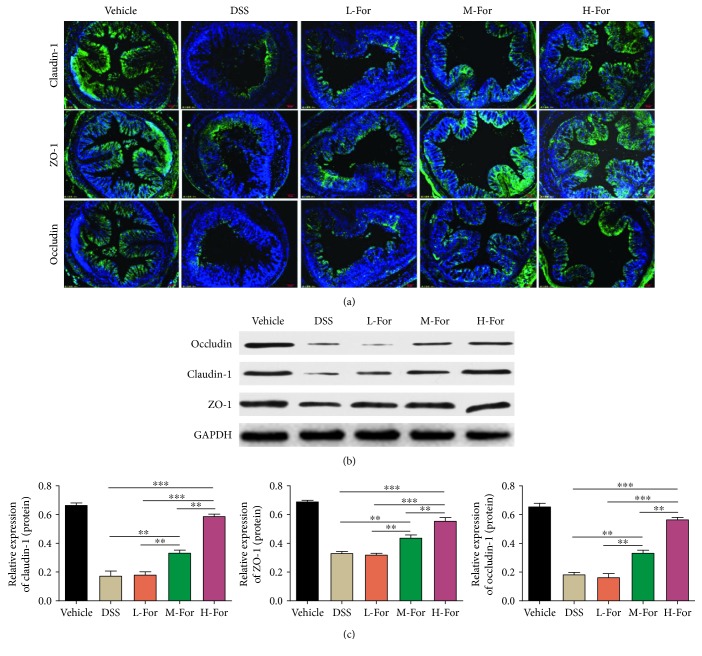
For relieved DSS-induced colonic epithelial tight junction destruction in mice. (a) Representative immunofluorescence images for claudin-1 (Green), occludin (Green), and ZO-1 (Green) in the colonic tissue. (b, c) Protein levels of claudin-1, occludin, and ZO-1 in the colon tissues were analyzed by western blotting. ^∗∗^ *p* < 0.01, and ^∗∗∗^ *p* < 0.001.

Tight junction (TJ) is an important structure foundation that maintains the mechanical barrier functions between intestinal mucosa epithelial cells and plays a critical role in the intestinal epithelial barrier integrity [25, 26]. We observed that the expressions of tight junction proteins, such as claudin-1, occludin, and ZO-1 remarkably reduced in the colonic tissues of DSS-induced colitis mice by adopting WB and immunofluorescence methods, and as we expected, the reduced expressions of tight junction proteins were in positive correlation with the integrity of intestinal epithelial barrier structure. Unsurprisingly, after formononetin administration, the reduced expressions of claudin-1, occludin, and ZO-1 proteins were counteracted (Figures 3(a)–3(c)), which provided another powerful evidence for the protective effects of formononetin on mice colitis.

### 3.4. Formononetin Inhibited NLRP3 Pathway in Mice Colonic Epithelial Cells

**Figure 4:**
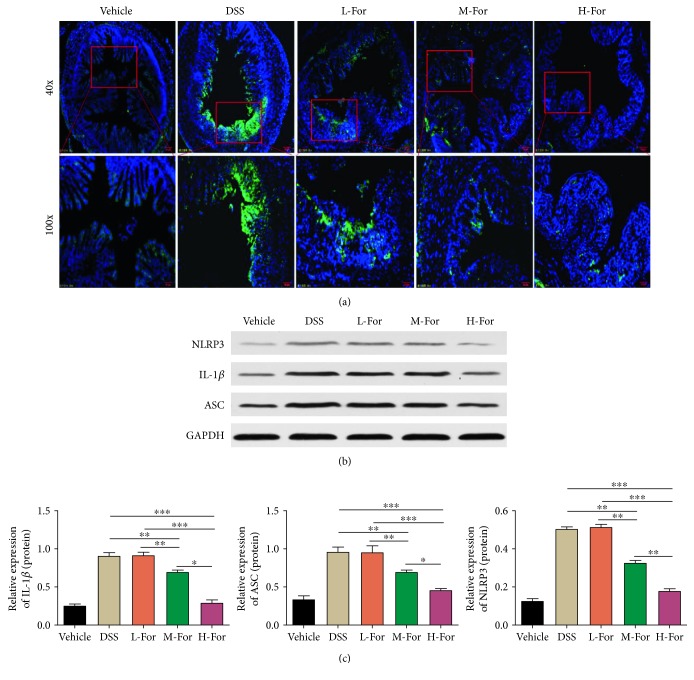
For inhibited NLRP3 pathway in mice colonic epithelial cells. (a) Representative immunofluorescence images for NLRP3 (Green) in the colonic tissue. (b, c) Protein levels of NLRP3, ASC, and IL-1*β* in the colon tissues were analyzed by western blotting. ^∗^ *p* < 0.05, ^∗∗^ *p* < 0.01, and ^∗∗∗^ *p* < 0.001.

Previous studies suggested that NLRP3 inflammasome pathway played an important role in colonic tissue injury in acute colitis in mice [17, 18, 27]. In this study, by using immunohistochemical staining, we firstly detected that the expression of NLRP3 in colonic tissue was elevated in DSS-induced colitis and formononetin reduced the activation of NLRP3 pathway (Figure 4(a)). Next, we observed that the expressions of NLRP3, IL-1*β*, and ASC proteins in colonic tissue were increased, which suggested that the NLRP3 inflammasome pathway was activated in the pathogenesis of colitis in mice. As we expected, formononetin administration reduced the expressions of NLRP3, IL-1*β*, and ASC significantly (Figures 4(b) and 4(c)), indicating that formononetin could exert the protective effect on colonic tissue injury by inhibiting the activation of NLRP3 pathway.

### 3.5. Formononetin Protected against Colonic Epithelial Tight Junction Disruption and Inhibited NLRP3 Pathway In Vitro

**Figure 5:**
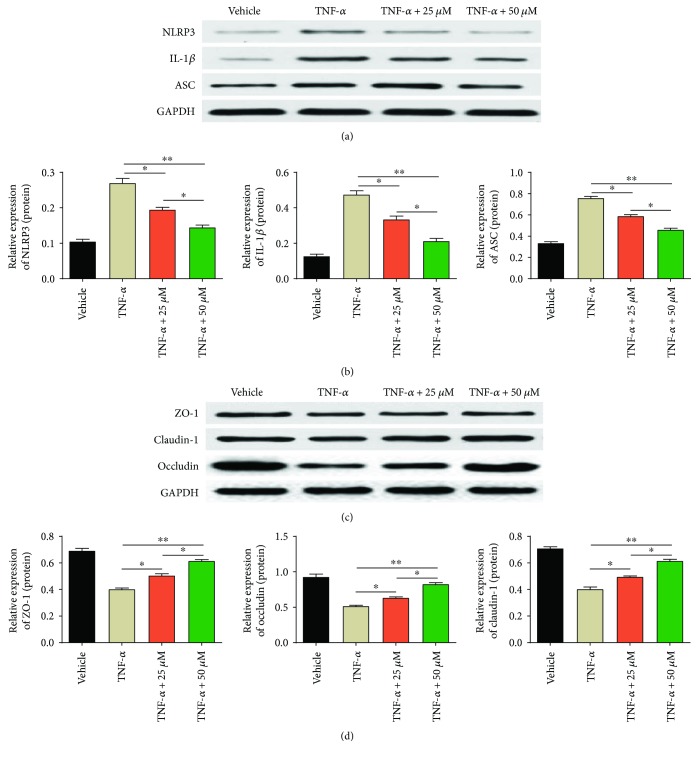
For protected against colonic epithelial tight junction injury and inhibited NLRP3 pathway in vitro. (a, b) Protein levels of NLRP3, ASC, and IL-1*β* and (c, d) claudin-1, occludin, and ZO-1 were analyzed by western blotting. ^∗^ *p* < 0.05 and ^∗∗^ *p* < 0.01.

In order to further explicit the protective effects of formononetin on acute colitis, the colonic cell line HCT-116 was employed to carry out the in vitro experiment. Firstly, we tested the cell viability of HCT-116 cells with TNF-*α* and formononetin treatment (Supplementary Figure S1, A-B), and the results showed that formononetin exerted no toxicity to HCT-116 while TNF-*α* dose-dependently reduced the cell viability of HCT-116 cells. According to previous literature [21], TNF-*α* (100 ng/ml) was used to establish the colonic cell injury model in vitro experiment. Next, after treatment with TNF-*α* and formononetin (25 *μ*M, 50 *μ*M), the expressions of tight junction proteins (claudin-1, occludin, and ZO-1) and NLRP3 inflammasome pathway (NLRP3, IL-1*β*, and ASC) were detected by WB methods. Unsurprisingly, formononetin protected the colonic cells from injury by the increased expressions of claudin-1, occludin, and ZO-1 (Figures 5(a) and 5(b)) together with the decreased expressions of NLRP3, IL-1*β*, and ASC (Figures 5(c) and 5(d)).

### 3.6. NLRP3 Inhibitor MCC950 Could Eliminate the Protective Effect of H-Dose Formononetin on Acute Colitis in Mice

**Figure 6:**
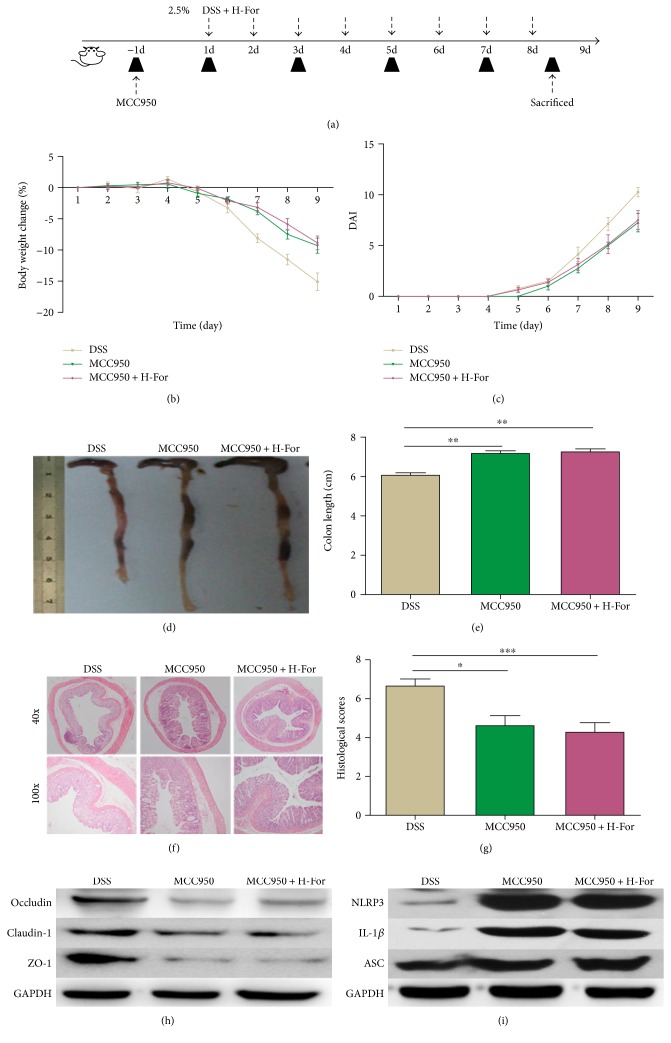
NLRP3 inhibitor MCC950 eliminated the protective effect of H-For on acute colitis in mice. (a) The experimental protocol with For and MCC950 in acute colitis model. (b) Body weights of mice and (c) disease activity index (DAI) during the disease process. (d) Morphological changes in the mice colons, (e) variations of colon length of mice, (f) representative HE staining, and (g) histological scores of colonic tissue. (h) Protein levels of claudin-1, occludin, and ZO-1 and (i) NLRP3, ASC, and IL-1*β* were analyzed by western blotting. ^∗^ *p* < 0.05, ^∗∗^ *p* < 0.01, and ^∗∗∗^ *p* < 0.001.

To further identify the underlying mechanisms of formononetin on acute colitis in mice, according to the above results, MCC950, the NLRP3-specific inhibitor [28], and high-dose formononetin (100 mg/kg) were adopted to carry out the following experiment. The experimental protocol with formononetin and MCC950 in acute colitis model was shown in Figure 6(a); MCC950 (50 mg/kg) was injected intraperitoneally every other day to inhibit the NLRP3 activity of mice. Similar to the previous literature results [18], MCC950 could significantly alleviate the loss of body weight, reduce the DAI score, and relieve the pathological injury of colon in mice, while administration of H-dose formononetin and MCC950 together failed to show synergetic effects, which meant that the protective effects of alleviating the loss of body weight, reducing the DAI score, and relieving the pathological injury of colon in mice showed no significant difference between H-dose formononetin + MCC95 group and MCC950 group (Figures 6(b)–6(g)). In addition, our results showed that the expressions of NLRP3, IL-1*β*, and ASC were reduced while the expressions of claudin-1, occludin, and ZO-1 were enhanced in mice colonic tissues after MCC950 administration, but the expressions of above proteins failed to show significant difference between H-dose formononetin + MCC95 group and MCC95 group (Figures 6(h) and 6(i), Supplementary Figure S3). All these findings indicated that formononetin exerted protective effects on DSS-induced acute colitis in mice via inhibiting NLRP3 inflammasome pathway.

## 4. Discussion

In this study, we for the first time verified that formononetin could alleviate the DSS-induced acute colitis in mice by inhibiting NLRP3 pathway. Ulcerative colitis (UC) is a kind of nonspecific inflammatory bowel disease, of which the main pathological features present as the inflammatory responses and ulceration formations in the mucosa and submucosa of the rectum or colon. Patients usually present with abdominal pain, bloody stool, and diarrhea as the main symptoms, in whom the active and remission stages often emerged in turn and eventually form a recurrent chronic disease course. DSS was used for the first time to establish the classical acute colitis model in rodents by Ohkusa in 1985 [29]. This novel DSS-induced colitis model was easy to prepare, economical, and could replicate the pathogenesis of human colitis suitably and accurately, so DSS-induced model was one of the most commonly used animal model for colitis studies [30, 31]. In this study, we adopted 2.5% DSS to induce a stable acute colitis mice model.

This present study focused on the protective effects of formononetin on DSS-induced colitis in mice. Previous studies have shown that 20 mg/kg formononetin could effectively protect against lipopolysaccharide-induced acute lung injury [7] and ameliorate blood glucose levels in alloxan-induced hyperglycemia in mice [32]; Jin et al. revealed that formononetin (50 mg/kg or 100 mg/kg) dose-dependently mitigated the acetaminophen-induced hepatotoxicity in mice [33]. Therefore, we selected gradient doses of formononetin (25 mg/kg, 50 mg/kg, and 100 mg/kg) for animal experiments. Fortunately, the results showed that formononetin alleviated the inflammatory responses in mice colitis in a dose-dependent manner, which was confirmed by histopathological manifestation, the length of colon, and the clinical symptoms of mice. In addition, formononetin administration reduced the infiltration of neutrophils and macrophages into the colonic tissues. Collectively, these results suggested that formononetin could mitigate the inflammatory responses of mice colitis effectively.

Tight junction destruction plays a key role in the development and progression of IBD [25, 26]. In previous animal experiment [34], it has been found that the congenital epithelial cell tight junction protein knockout mice appeared with intestinal pathological changes which was similar with that of IBD after birth, implying that claudins were involved in the pathogenesis of IBD. Moreover, by adopting immunofluorescence and western blot, it could be observed that the expressions of claudin-1, occludin, and ZO-1 were downregulated in DSS-induced acute colitis model in rats [35, 36]. A large number of studies have shown that restoring and maintaining intestinal mucosal barrier function were beneficial to improve the defensive function of intestinal mucosa, promote disease remission, and reduce relapse times of IBD [37, 38]. Our result showed that the expressions of epithelial cell tight junction proteins claudin-1, occludin, and ZO-1 were reduced remarkably in DSS-induced colitis in vivo and in TNF-*α*-induced cell injury model in vitro, while after the administration of formononetin, we found that formononetin increased the expressions of claudin-1, occludin, and ZO-1 significantly, suggesting that formononetin could protect the colonic mucosal integrity and maintain the colonic epithelial barrier function of colitis.

NLRP3 presents as the most important member of pattern recognition receptor NLR family and recognizes the danger signals released by cellular pathogens or cells themselves [39], and it can combine with ASC to form a multimeric protein complex which is named NLRP3 inflammasome. Excessive activation of NLRP3 inflammasome has been proven to play a key role in a variety of inflammatory diseases, such as diabetes [40], Alzheimer's disease [41], and atherosclerosis [42]. Similarly, NLRP3 inflammasome also plays a critical role in the pathogenesis of IBD [17, 18]. Previous studies indicated that the expressions of NLRP3, ASC, and caspase-1 were remarkably elevated in colonic tissues of colitis both in patients and in animal models; meanwhile, the disease severities of colitis in NLRP3, ASC, and caspase-1 knockout mice tended to be much relieved in comparison with the wild-type mice, suggesting that NLRP3 inflammasome pathway exerted a great effect on the development and progression of colitis. More importantly, the inhibition of NLRP3 pathway could mitigate the disease severity of colitis [18]. In this study, formononetin showed great inhibitory effect on the expressions of NLRP3, ASC, and IL-1*β* and reduced the secretion of IL-1*β* significantly in colonic epithelial cells in vivo and vitro. Surprisingly, the NLRP3-specific inhibitor MCC950 could alleviate the DSS-induced acute colitis in mice; however, in the basis of administrating MCC950 to inhibit the activation of NLRP3, we failed to observe the protective effects of formononetin on acute colitis additionally. Based on the above results, our study for the first time demonstrated the inhibitory effect of NLRP3 inflammasome pathway by formononetin.

## 5. Conclusion

Through this study, we concluded that formononetin could protect colonic epithelial cells from injury to relieve the disease severity of colitis in mice via inhibition of NLRP3 inflammasome pathway. Formononetin may be a promising strategy for the clinical prevention and treatment of IBD in the future.

## Supplementary Materials

Supplementary Materials Figure S1: Chemical structure of formononetin. Figure S2: Cell viability was measured by MTS assay in different concentration of TNF-*α* (A) and formononetin (B) on HCT-166 cells. Data are shown as relative cell viability (mean % ± S.E. bar) as compared with that in control (*n* = 5). Figure S3: NLRP3 inhibitor MCC950 eliminated the protective effect of H-For on acute colitis in mice. Protein levels of (A) claudin-1, occludin, and ZO-1 and (B) NLRP3, ASC, and IL-1*β* were analyzed by western blotting. ^∗^ *p* < 0.05, ^∗∗^ *p* < 0.01.
