# Corrigendum to “Formononetin Administration Ameliorates Dextran Sulfate Sodium-Induced Acute Colitis by Inhibiting NLRP3 Inflammasome Signaling Pathway”

**DOI:** 10.1155/2018/9878120

**Published:** 2018-09-06

**Authors:** Dacheng Wu, Keyan Wu, Qingtian Zhu, Weiming Xiao, Qing Shan, Zhigang Yan, Jian Wu, Bin Deng, Yan Xue, Weijuan Gong, Guotao Lu, Yanbing Ding

**Affiliations:** ^1^Department of Gastroenterology, Affiliated Hospital of Yangzhou University, Yangzhou University, Yangzhou, China; ^2^Laboratory of Gastroenterology, Affiliated Hospital of Yangzhou University, Yangzhou University, Yangzhou, China; ^3^Department of Immunology, School of Medicine, Yangzhou University, Yangzhou, China

In the article titled “Formononetin Administration Ameliorates Dextran Sulfate Sodium-Induced Acute Colitis by Inhibiting NLRP3 Inflammasome Signaling Pathway” [1], the protein labels in Figures 6(h) and 6(i) were reversed and should be corrected as follows:

## Figures and Tables

**Figure 6:**
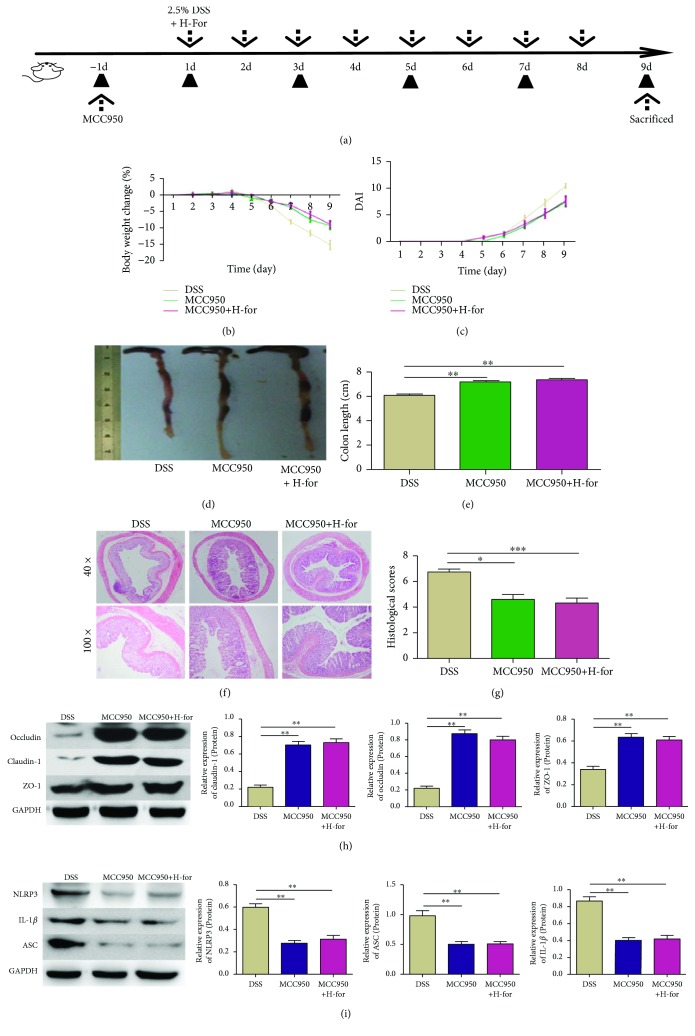
NLRP3 inhibitor MCC950 eliminated the protective effect of H-For on acute colitis in mice. (a) The experimental protocol with For and MCC950 in an acute colitis model. (b) Body weights of mice and (c) disease activity index (DAI) during the disease process. (d) Morphological changes in the mouse colons, (e) variations of colon length of mice, (f) representative HE staining, and (g) histological scores of colonic tissue. (h) Protein levels of claudin-1, occludin, and ZO-1 and (i) NLRP3, ASC, and IL-1*β* were analyzed by western blotting. ^∗^ *p* < 0.05, ^∗∗^ *p* < 0.01, ^∗∗∗^ *p* < 0.001.
