# Supplementary material for: Formononetin Administration Ameliorates Dextran Sulfate Sodium-Induced Acute Colitis by Inhibiting NLRP3 Inflammasome Signaling Pathway
Source: Mediators Inflamm. 2018 Jan 8;2018:3048532. doi: 10.1155/2018/3048532 (PMC5817291; doi:10.1155/2018/3048532)
Supplement: Supplementary Materials — Figure S1: Chemical structure of formononetin. Figure S2: Cell viability was measured by MTS assay in different concentration of TNF-α (A) and formononetin (B) on HCT-166 cells. Data are shown as relative cell viability (mean % ± S.E. bar) as compared with that in control (n = 5). Figure S3: NLRP3 inhibitor MCC950 eliminated the protective effect of H-For on acute colitis in mice. Protein levels of (A) claudin-1, occludin, and ZO-1 and (B) NLRP3, ASC, and IL-1β were analyzed by western blotting. ∗ p < 0.05, ∗∗ p < 0.01. [file 3048532.f1.pdf]

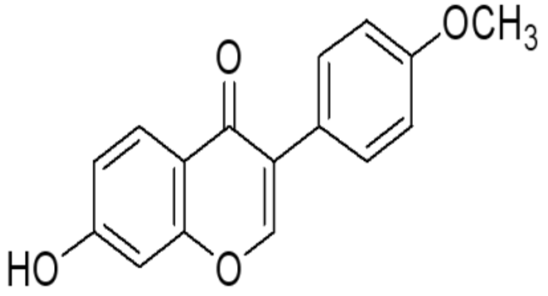

Supplementary Figure S1. Chemical structure of formononetin.

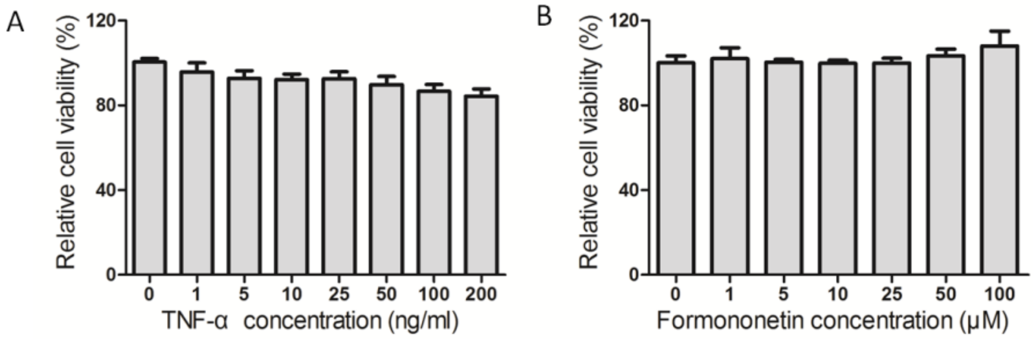

Supplementary Figure S2. Cell viability was measured by MTS assay in different concentration of TNF-α (A) and Formononetin (B) on HCT-166 cells. Data are shown as relative cell viability (mean % ± S.E. bar) as compared with that in control (n = 5).

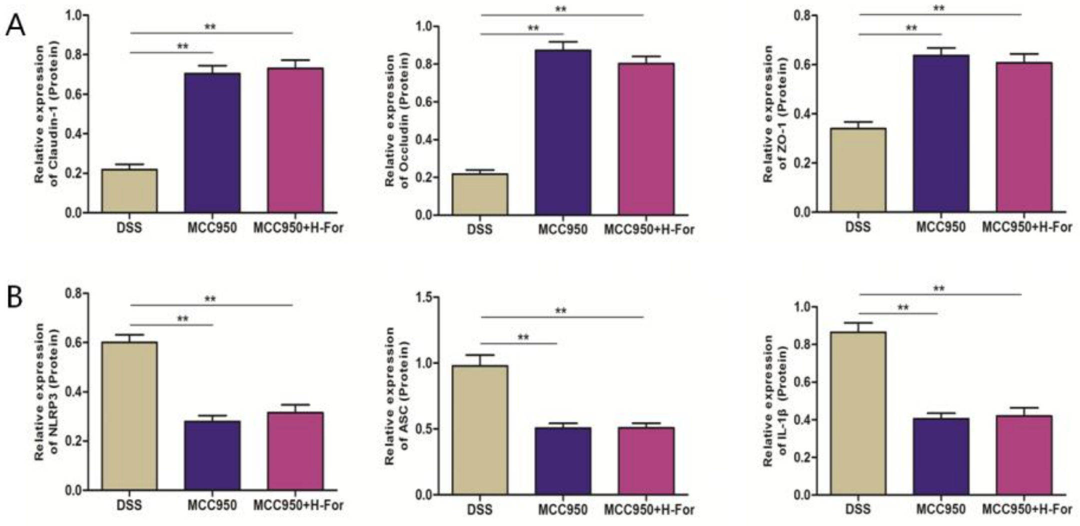

Supplementary Figure S3. NLRP3 inhibitor MCC950 eliminated the protective effect of H-For on acute colitis in mice. Protein levels of (A) Claudin-1,Occludin and ZO-1 and (B) NLRP3, ASC, and IL-1β were analyzed by western blotting. *p<0.05, **p<0.01.
